# Recycling paper waste into structural cellulose composites with enhanced mechanical and thermal performance

**DOI:** 10.1038/s41598-026-43032-7

**Published:** 2026-03-21

**Authors:** Marcin Szczepanski, Ahmed Manguri

**Affiliations:** 1 Faculty of Civil and Environmental Engineering, Gdansk University of Technology, 80-223, Gdansk, Poland; 2 Civil Engineering Department, College of Engineering, University of Raparin, Rania, Kurdistan Region Iraq

**Keywords:** Composite, Cellulose, Mechanical properties, Thermogravimetric, Recycling paper, Engineering, Environmental sciences, Materials science

## Abstract

Recycling post-consumer cellulose waste for use in construction materials has largely been limited to board-type composite materials. While the wood-panel market is dominated by OSB and MDF, there is limited research concerning structural boards produced directly from recycled paper waste through simple processing methods. This work examines the potential to produce rigid polyurethane–cellulose composite boards using ground waste newspapers as a filler material without a catalyst. Composite boards containing 10–50 wt.% cellulose were manufactured by hot pressing and evaluated for tensile and compressive strengths, Charpy impact testing, thermogravimetric analysis, dynamic mechanical analysis, and water vapour permeability. The modulus of elasticity increased nearly three times from 10% cellulose content to 50% cellulose content due to the increased cellulose content. The maximum compressive strength was achieved at 30% cellulose content. Impact strength was not significantly impacted by filler content, resulting in consistent values for all compositions. Thermogravimetric analysis indicated that, with increasing cellulose fraction, thermal stability improved (e.g., the peak decomposition temperature (PDT) increased). Dynamic mechanical analysis results confirmed that, for boards with high cellulose content, stiffness and energy dissipation increased with each incremental increase in cellulose content. The results of the study indicate that paper recycling waste can be processed into a mechanically stable material, Polyurethane-Cellulose Boards, for use in many non-load-bearing applications within the construction sector. Using these boards represents a viable and scalable means of converting cellulose waste into a usable product in the construction industry.

**Supplementary Information:**

The online version contains supplementary material available at 10.1038/s41598-026-43032-7.

## Introduction

Many construction products, such as wood-based boards, are widely available. The most popular options are oriented strand board (OSB)^1^ and medium-density fiberboard (MDF)^2^. A common trait is that they are made from new raw materials sourced from cutting down trees^3^. OSB composite boards are produced from chips derived from cutting round wood (usually pine) and are bonded with a synthetic resin binder^4,5^. In contrast, MDF boards are crafted from ground wood fibers^6^, resulting in a more uniform structure compared to chips. In a study by Nguyen et al.^7^ a wood-based panel was produced based on waste wood.

One potential approach is to utilize waste, which, after suitable processing, can be transformed into boards with properties that make them alternatives to well-known OSB and MDF boards. Excessive waste production is one of the most significant problems facing the modern world. Due to the ongoing development of civilization, the demand for consumer goods is continuously increasing, and consequently, the amount of waste generated is also rising^8^. These are substances that have ceased to fulfill the original purposes for which they were produced. Waste can take various forms, including solid^9,10^, liquid^11^, or gaseous^12^, and it originates from a range of sources, such as households, industry, and agriculture. The most common type is municipal waste from households, generated by non-industrial human activity. This includes waste such as paper, plastic, glass, metals, biodegradable materials, and mixed waste.

Researchers conducted tests to evaluate the mechanical properties of rigid polyurethane foams (RPUFs) incorporating various concentrations of cellulose nanocrystals (CNCs). The results indicated that RPUFs containing 3% CNC exhibited optimal mechanical performance and cellular morphology, whereas further addition of 5% CNC resulted in decreased compressive strength due to agglomeration^13^. In another study, Sezgin et al.^14^ produced high-value composite materials from textile and packaging waste. They reported that denim waste fabric is utilized as reinforcement, with polypropylene and polyethylene caps serving as the matrix. In a study Ibrahim et al.^15^ outlined the environmental impact of burning agricultural waste and proposed upcycling it into wood composites in an eco-friendly manner using Aspergillus niger mycelium. The authors noted the efficiency of gamma irradiation and autoclaving as preliminary treatments to enhance adhesion in wood composites. They also found improved tensile strength and water resistance for EPS-induced Aspergillus niger-treated composites at 25%.

Liew et al.^16^ conducted an analysis of the physical and mechanical properties of seaweed, starch, and wood-plastic composite (WPC) blends with high-density polyethylene (HDPE) and Acacia mangium wood powder. The study involved experimenting with different seaweed-to-starch ratios as binders for the particleboard. Cellulose is a raw material commonly used in the paper industry for the production of paper, cardboard, paperboard, etc.^17^, as also highlighted in comprehensive reviews on cellulose nanocrystals and nanofibers^18^.Cellulose Nanofibers (CNF) from Ramie were used to strengthen bio-polymer matrix materials, such as hybrid Cassava Starch composites. Thus, CNF provides an opportunity to improve the strength and stiffness of bio-polymer composites by reinforcing them at the nanoscale using the aforementioned mechanism^19^.While studies exist on wood-based panels and natural fiber composites^20^. Research on the mechanical performance of boards fabricated from post-consumer cellulose waste (e.g., ground newspaper) with polyurethane binders remains limited, particularly regarding their use as replacements for commercial-oriented strand board (OSB) or medium-density fiberboard (MDF) panels in the construction industry.

The pyrolysis characteristics of various feedstock samples can be examined using thermogravimetric analysis (TGA). TGA is a crucial tool for studying the thermal properties of a substance during heating, including thermoregulation dynamics and kinetics. Exploring the potential of waste biomass for sustainable bioenergy production paves the way for a circular bioeconomy and helps reduce our heavy reliance on nonrenewable energy sources. Escalante et al.^21^ provided comprehensive insight into the extensive use of TGA in supporting research and development of pyrolysis from different waste biomass sources. The thermal properties of various biomass wastes, as determined by TGA, were also discussed. Zhang et al.^22^ investigated the pyrolysis kinetics of cellulose, hemicellulose, and lignin using thermogravimetric analysis and distributed activation energy models. While a single Gaussian-DAEM accurately described cellulose decomposition, a double Gaussian-DAEM was required to reliably capture the pyrolysis behavior of hemicellulose and lignin.

This study describes the development and characterization of a novel board-type composite material fabricated exclusively from cellulose-based paper waste and a polyurethane binder, without the use of additive catalysts. Emphasizing the significance of sustainability and its industrial relevance, this research examines the potential use of the material as a substitute for wood in construction, cladding, and insulation. A comprehensive series of standard mechanical and physical tests, including axial tensile, compressive, and Charpy impact tests, as well as thermographic analysis, water vapor permeability, and DMA tests, was conducted to evaluate the composite material’s performance.This study hypothesizes that increasing the cellulose content in the paper–polyurethane composite improves its mechanical performance while maintaining adequate ductility. The aim is to evaluate the composite’s mechanical behavior and compare it with OSB and MDF boards.The results were compared with those of conventional commercial materials, yielding insights into the composite’s feasibility as a sustainable, viable replacement in the built environment. On top of that, the proposed structural cellulose composites achieve reduced embodied carbon while offering mechanical and thermal performance appropriate for low-carbon construction applications.

Paper waste was recycled and reused as a sustainable filler for asphalt mixtures^23^ and in stone mastic asphalt^24^ and brick production^25^. This work builds upon previous studies on polyurethane nanocomposites and the use of cellulose-based materials in polymer technology. Prior research has demonstrated the unique role of nanofillers in enhancing the performance of polyurethane systems^26^ as well as the feasibility of incorporating biopolymers from waste biomass into rigid foams^27^. Moreover, the use of recycled newspaper-derived polyols in board materials remains underexplored, particularly in the context of catalyst-free synthesis and mechanical optimization^28^. Therefore, this study aims to develop rigid polyurethane boards based on newspaper-derived polyols without additional catalysts and to evaluate their potential for load-bearing and impact-resilient applications, with a focus on sustainable solutions for the construction sector. The objective of this study is not to propose a direct structural replacement for OSB or MDF, but to explore the feasibility of recycled cellulose–polyurethane boards as sustainable alternatives for specific building applications where full structural equivalence is not required.

## Materials and methods

The following section describes the procedure for producing composite boards from cellulose waste. Their composition and the activities carried out in the laboratory will be discussed.

### Material composition

The following raw materials were used to obtain composite polyurethane-cellulose boards:

- Cellulose filler in the form of crushed newspaper waste with different percentages in the sample mass;
- Petrochemical polyol Rokopol RF551 (LOH = 420 KOH/g) and M6000 (LOH = 20 KOH/g) supplied by PCC Rokita SA (Brzeg Dolny, Poland);
- Polymeric methylene diphenyl diisocyanate (pMDI, 31% free NCO groups)

The raw materials included in the composites were grouped as follows:

- Component A: polyol M6000 and polyol RF551 in a ratio of 20:80
- Component B: pMDI.

The ratio of Component A to Component B was kept at 1:1 for all samples, and no catalyst was included in the composite formulation. The cellulose filler content, represented by the symbol C, ranged from 10 to 50% by mass, in increments of 10%. The remaining material consisted of polyurethane, created by mixing Components A and B. Sample identifiers were assigned according to the cellulose content (C), as outlined in Table 1. The specific amounts of each raw material used in preparing the samples are listed in Table 2.

**Table 1:** Designations of polyurethane-cellulose samples.

Sample designation	Percentage of filler	Percentage of polyurethane
C	%	%
10	10	90
20	20	80
30	30	70
40	40	60
50	50	50

**Table 2:** Composition of polyurethane-cellulose samples.

Component		Quantity g % share of natural filler fraction				
		10%	20%	30%	40%	50%
Component A	M6000	18.70	16.70	14.60	12.50	10.41
	RF551	75.00	66.80	58.35	50.00	41.75
Component B	pMDI	77.20	68.70	60.00	51.50	42.90
Filler		19.00	38.00	57.00	76.00	95.0

### Sample production process

**Fig. 1:**
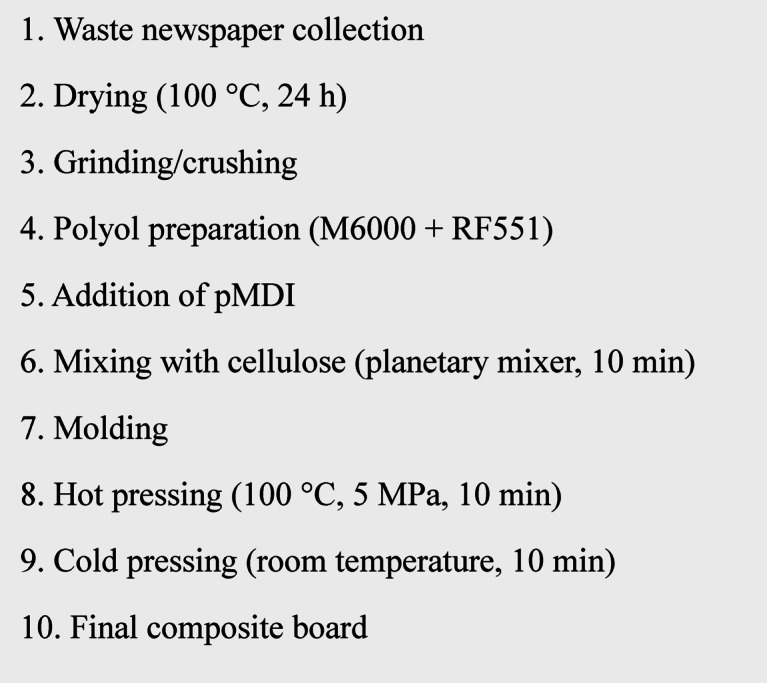
A schematic illustration of the composite manufacturing and board fabrication process.

Before composite fabrication, cellulose waste was dried at 100 °C for 24 h. The required amounts of Components A and B were then individually measured. The dried cellulose filler was placed into the bowl of a planetary mixer. Separately, M6000 and RF551 were pre-mixed in a laboratory cup for 30 s using a laboratory rod. Following that, pMDI was added to the cup with a syringe, and mixing continued for an additional 30 s. The resulting mixture was transferred to the planetary mixer and blended with the cellulose filler for 10 min. After blending, the composite was poured into a steel mold and hot-pressed at 100 °C and 5 MPa for 10 min. Cold pressing at room temperature under the same pressure was performed to stabilize the shape and cool the samples for an additional 10 min. The resulting composite plates were cuboidal, measuring 200 × 100 × 10 mm. For future testing, sample sizes were adjusted to the necessary dimensions for each procedure. A schematic illustration of the composite manufacturing and board fabrication process is shown Fig. 1.

### Tensile strength test

Axial tensile tests were conducted on cellulose–polyurethane composite specimens with cellulose contents ranging from 10 to 50% by weight. Composite plates were cut into five specimens per composition (≈10 × 10 × 200 mm) and labeled using the C.M. Specimens were labeled using the C.M. convention. Where C represents the cellulose level (ranging from 10 to 50% in increments of 10%), and M represents the specimen number (from 1 to 5). Prepared specimens are shown in Fig. 14 (Appendix). Due to cutting tolerances, cross-sectional dimensions were measured with a caliper, and the calculated areas are reported in Table 8 in the appendix. Tensile testing was performed using a ZwickRoell Z400 universal testing machine (400 kN capacity). Hydraulic clamping caused specimen damage; therefore, manually tightened jaws were used. The jaw separation was set to 120 mm. Axial strain was measured using an extensometer with a 50 mm gauge length.

Preliminary tests without an extensometer were conducted on one specimen per cellulose content (10.1%–50.1%) to characterize failure behavior and determine a safe activation limit for the extensometer. These tests also provided an initial estimate of the elastic modulus. Based on control specimens (Fig. 15 in the appendix), the extensometer disconnection threshold was set to 300 kN. All tests were performed under the following conditions: 120 mm jaw separation, 50 mm gauge length, 300 kN disconnection limit, 10 N preload, and 0.5 mm/min crosshead speed. Axial tensile testing was conducted in accordance with ISO 527–1 and ISO 527–2, with minor specimen geometry adaptations permitted for rigid polymer-based composite materials. A typical test configuration is shown in Fig. 16 (appendix). Representative fracture modes are illustrated in Figs. 17 and 18 in the appendix. Failure occurred near the specimen mid-length in all cases, except for specimen 30.3, which failed near the grips (Fig. 19 in the appendix).The sample size is commensurate with exploratory material characterization, but the statistical power to detect small group differences is limited.

### Axial compression test

For the compression tests, four cubic specimens were prepared for each cellulose content, with nominal dimensions of 10 × 10 × 10 mm. Due to cutting variability, cross-sectional dimensions were measured using a caliper, and the corresponding areas, A, were calculated. The measurement results are summarized in Table 9 (appendix).An initial force of 2 N was applied to ensure the samples were correctly positioned between the jaw linings. Subsequent compression tests were conducted on the samples. Based on the height of each cube, the machine software automatically converted the piston displacement into deformation, expressed as a percentage. It was observed that the material did not exhibit a distinct moment of failure. The cubes were gradually crushed, so it was decided to halt further compression once shortening reached 40–50%. Compressive testing was performed in accordance with ISO 604, which is commonly applied to rigid plastics and polymer-based composite materials under uniaxial compression. There were four specimens per composition due to limitations in specimen preparation. This means statistical significance should be viewed with some caution.

### Impact test

Impact resistance of the polyurethane–cellulose composites was evaluated using the Charpy impact test on a ZwickRoell HIT5.5P testing machine, in accordance with ISO 179–1 for polymer-based composite materials. Although not commonly applied to cellulose composites, the Charpy method was selected due to its suitability for assessing the dynamic brittleness and toughness of low-density boards under impact loading^29–31^.

All tests were conducted at room temperature. Specimens were V-notched to induce stress concentration at the impact location. During testing, a pendulum hammer released from a predefined height struck the notched specimen, and the absorbed fracture energy was determined from the difference in pendulum height before and after impact. Impact strength was calculated as the absorbed energy divided by the cross-sectional area at the fracture surface. A schematic of the Charpy test setup is shown in Fig. 20, while specimen preparation with the V-notch is illustrated in Fig. 21 (appendix).Testing was performed using a hammer corresponding to an impact energy of 2.0 J. The prepared test station is shown in Fig. 22 in the appendix. The machine automatically recorded the breaking energy for each specimen, after which fracture dimensions were measured using a caliper.Three samples of each composition were tested. This small sample size makes it difficult to rely on statistical testing results, and caution is necessary when interpreting the observed trend.

### Thermogravimetric analysis

Thermogravimetric analysis (TGA) was conducted on cellulose waste and cellulose–polyurethane composites with varying waste contents using a NETZSCH TG 209 F3 instrument. One representative sample was tested for each material composition. Due to instrument constraints, small sample masses (≈10 mg) were used, as summarized in Table 10 (appendix).All measurements were performed under a nitrogen (N₂) atmosphere using a dynamic heating program. Samples were heated from 35 °C to approximately 800 °C at a constant heating rate of β = 10 °C/min. Mass changes were continuously recorded using a high-precision thermobalance, with temperature measured by a thermocouple positioned adjacent to the sample.Dynamic TGA was employed, in which sample mass variation was measured as a function of temperature under a linear heating rate. The thermogravimetric (TG) curve and its first derivative (DTG) were used to evaluate thermal stability and decomposition behavior. Due to limitations in the thermogravimetric analysis equipment, only one representative sample per composition was evaluated. Therefore, the TGA results are presented qualitatively.The TG curve is defined as

1 $$G = f(T)$$

Hence, the DTG derivative curve is defined as:

2 $$\frac{dG}{{dT}} = f^{\prime } (T)$$

where T = β*t,

### Water vapor permeability

In this test, three different cellulose content levels were tested. For each of them, five samples (1–5) were tested. For each sample, the average thickness of four samples (M1-M4) was determined due to molding inaccuracies and thickness variations, as presented in Table 3 and Fig. 23 (appendix).

**Table 3:** Thickness in (mm) of samples with various cellulose contents.

Content %	10				30				50			
Samples	M1	M2	M3	M4	M1	M2	M3	M4	M1	M2	M3	M4
1	10.7	10.6	10.6	10.5	10.8	11.1	11	11	11	10.9	10.8	10.9
2	10.5	10.2	10.4	10.2	10.7	10,6	10.7	10.7	10,9	10.8	10.7	10.8
3	11	10.8	10.7	10.8	10.6	10.5	10.6	10.4	10.7	10.5	10.3	10.5
4	11.1	11.5	11.4	11.4	10.5	10.5	10.4	10.5	10.6	10.5	10.5	10.4
5	12	11.8	11.9	11.9	10.5	10.7	10.6	10.6	10.9	11	10.8	10.9

## Results and discussions

### Tensile strength test

**Fig. 2:**
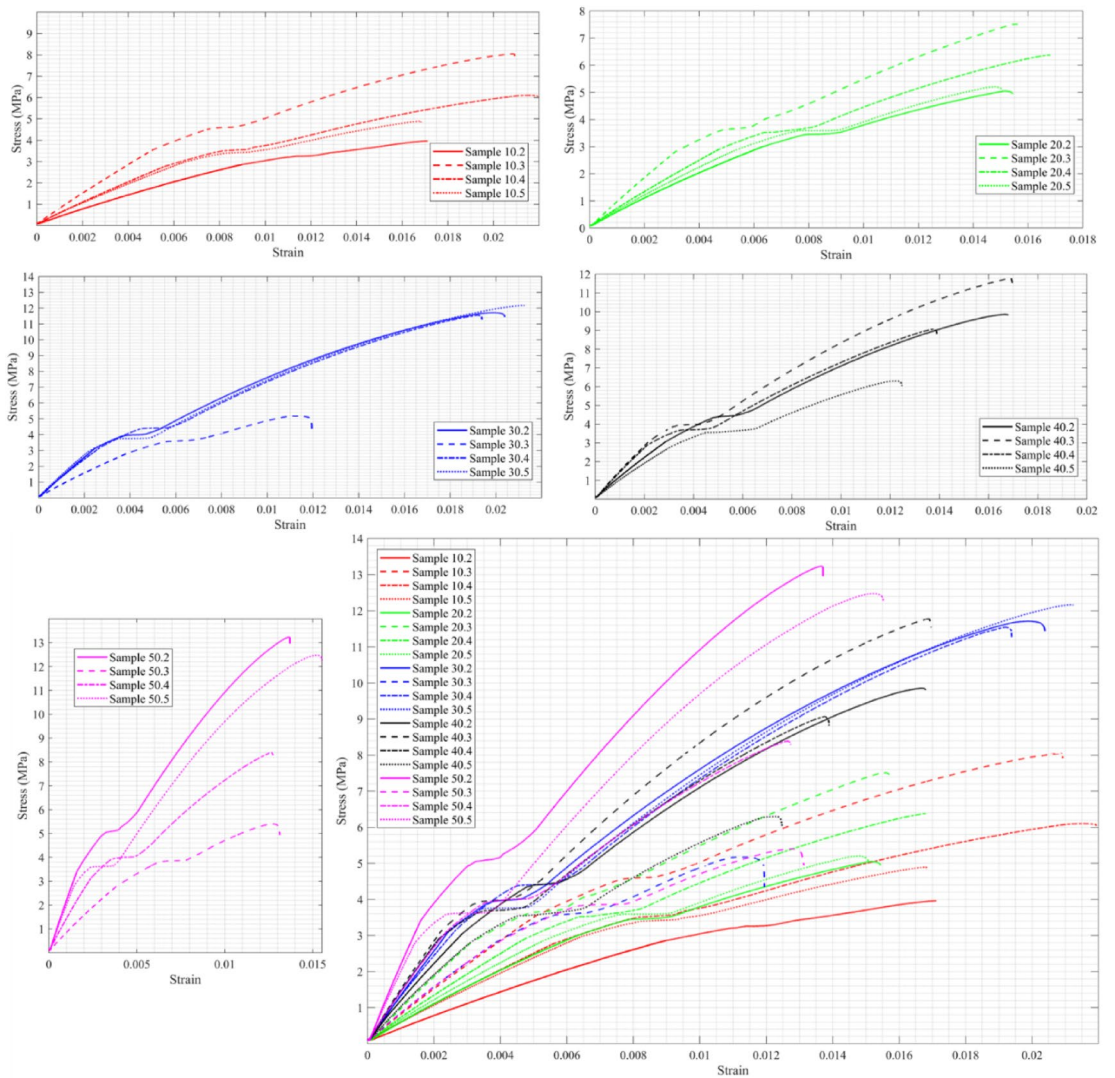
Stress–strain diagram of C10-C50, M2-M5, and all cases together under tensile stress.

This subsection presents and discusses the tensile stress–strain diagrams of the material with varying C content. Figure 2 illustrates the stress–strain diagrams of sixteen samples, ranging from C10 to C50 and M2 to M5, along with the combined results for all cases. It is evident that increasing the C ratio enhances the material’s strength. Regarding the material’s ductility, it appears to become more brittle as the C ratio increases; however, this does not apply to C = 30. When C = 50, the material withstands the highest stress, slightly over 13 MPa, and exhibits greater brittleness than in all other cases, with a maximum strain of 0.015.

**Fig. 3:**
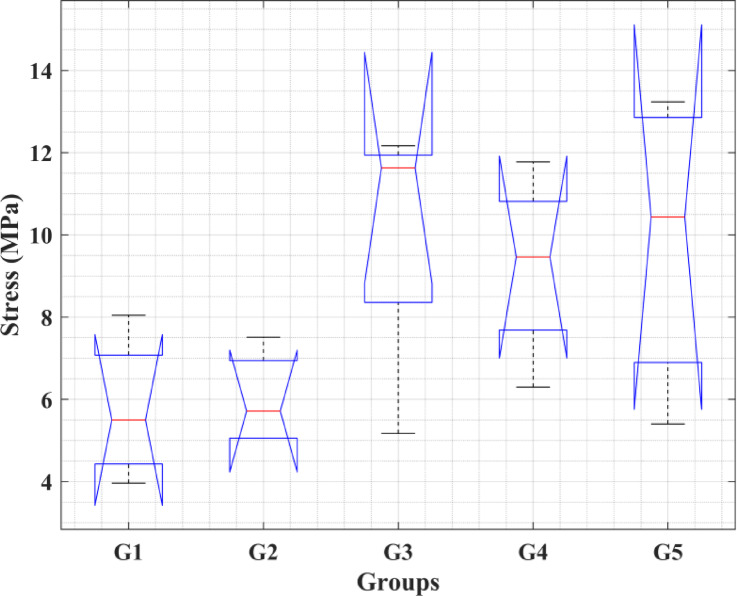
Kruskal–Wallis test for the tensile strength of the five groups.

A Kruskal–Wallis test was conducted to compare the distributions of the five groups, as shown in Fig. 3. While a tendency toward increased tensile strength with increasing cellulose content was observed, the Kruskal–Wallis test showed no significant differences between groups (p = 0.0801). Thus, this data can only be considered a tendency rather than proof of an increase in tensile strength. It should be noted that G1-G5 represent C10-C50, respectively. In other words, an increase in tensile strength with higher cellulose content is visually apparent; however, the Kruskal–Wallis test did not indicate statistically significant differences among the groups (p = 0.0801). Therefore, this trend should be interpreted as indicative rather than statistically confirmed.

#### Comparison with OSB

**Fig. 4:**
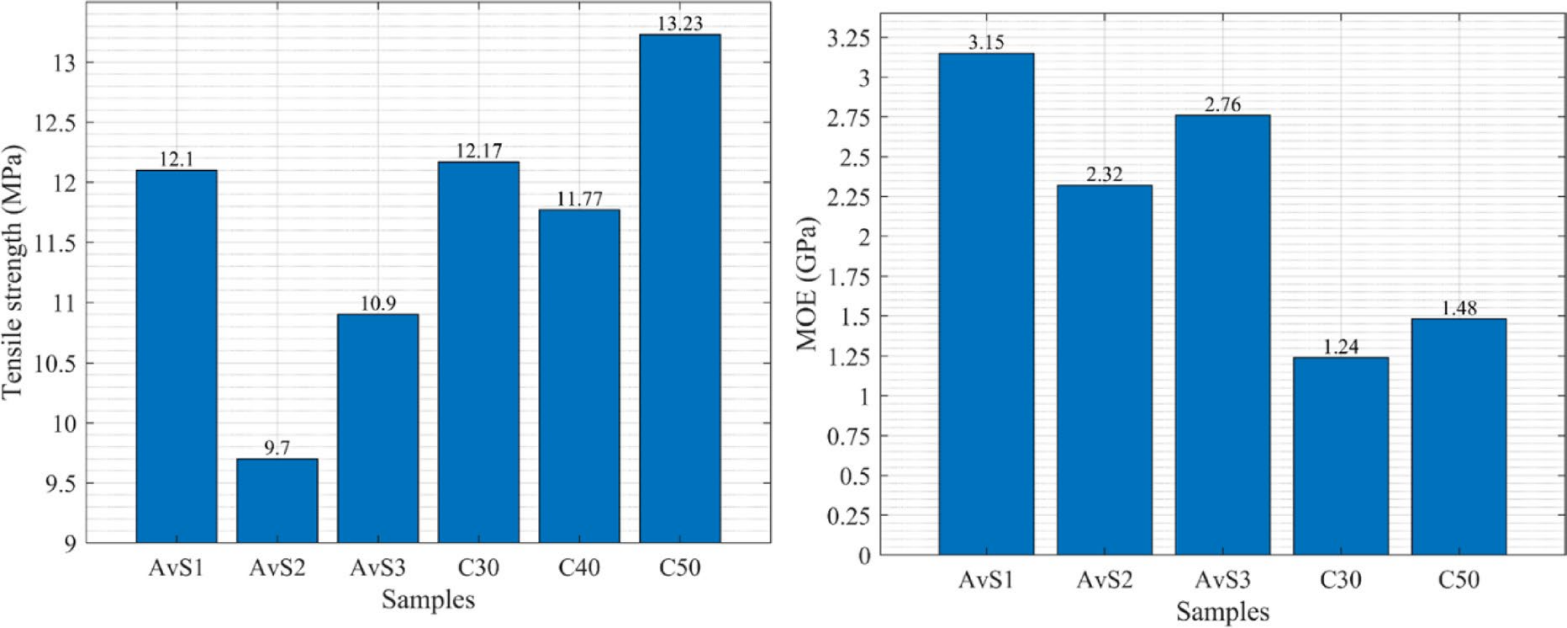
Comparison of the axial tensile strength and the MOE of C30-50 and OSB from^32^.

According to the results obtained by Chen and He^32^, the tensile strength of the material proposed in this study exceeds that of the OSB manufactured in their research. Figure 4 illustrates the results of the tensile strength from three different samples in Chen and He^32^ study alongside the C30-50 samples from the current research. AvS1-S3 represents the average of several samples across three groups. The figure shows that both C30 and C50 exhibit improved tensile-stress resistance compared to the previous study.

#### Determination of modulus of elasticity (MOE)

**Fig. 5:**
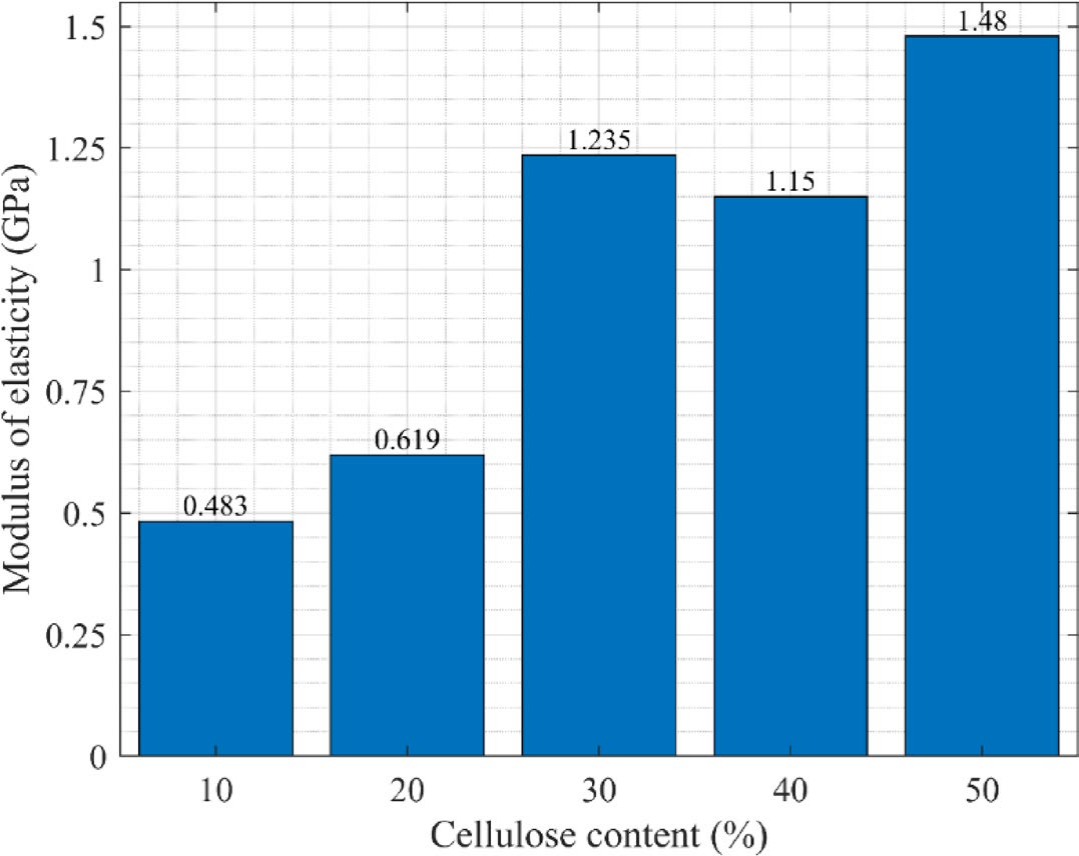
MOE is based on the tensile stress ofthe samples with varying cellulose contents.

The results of the tensile test, conducted on a tensile testing machine that measured strain with an extensometer, will be used to determine the MOE for each tested sample. Hooke’s law was applied for this purpose; the values of stress and strain were read from the most linear initial section of the graph. The tests were calculated according to the following formula, and the average MOE was computed for each cellulose content, as presented in Fig. 5.

3 $$E = \frac{\sigma }{\varepsilon }$$

The average MOE is shown in Fig. 5, indicating that an increase in cellulose content in the samples led to greater stiffness and tear strength. More specifically, a five-fold increase in cellulose content led to approximately a three-fold increase in MOE. The relationship is not perfectly linear, as depicted in Fig. 5; the 30% sample unexpectedly exhibited higher stiffness compared to the 40% sample.Comparable mechanisms for increasing stiffness have also been observed in other fiber-reinforced polymer composites intended for lightweight structural applications, such as hybrid hemp, pineapple, and glass fiber epoxy composites, in which the reinforcing pattern is the dominant factor in load transfer effectiveness. Unlike these other types of fiber composites, the polyurethane-cellulose composites described in this study contain randomly dispersed recycled cellulose particles, which have a correspondingly lower absolute stiffness but also simpler processing and greater potential for scalability as boards^33^.

The increased use of waste-derived cellulose may increase tensile stiffness, although the lack of statistical evidence for these trends precludes a final conclusion.These results imply that increased use of cellulose derived from waste materials could improve mechanical properties; however, given the variability and small sample size, further verification is necessary to confirm this trend.

The MOE of C50 in this study is lower than that of the study by Chen and He^32^, as shown in Fig. 4; however, their tensile strengths are opposite. These results indicate that the cellulose-based boards experience greater strains due to their greater ductility than the OSB produced by Chen and He^32^.

### Axial compression test

**Fig. 6:**
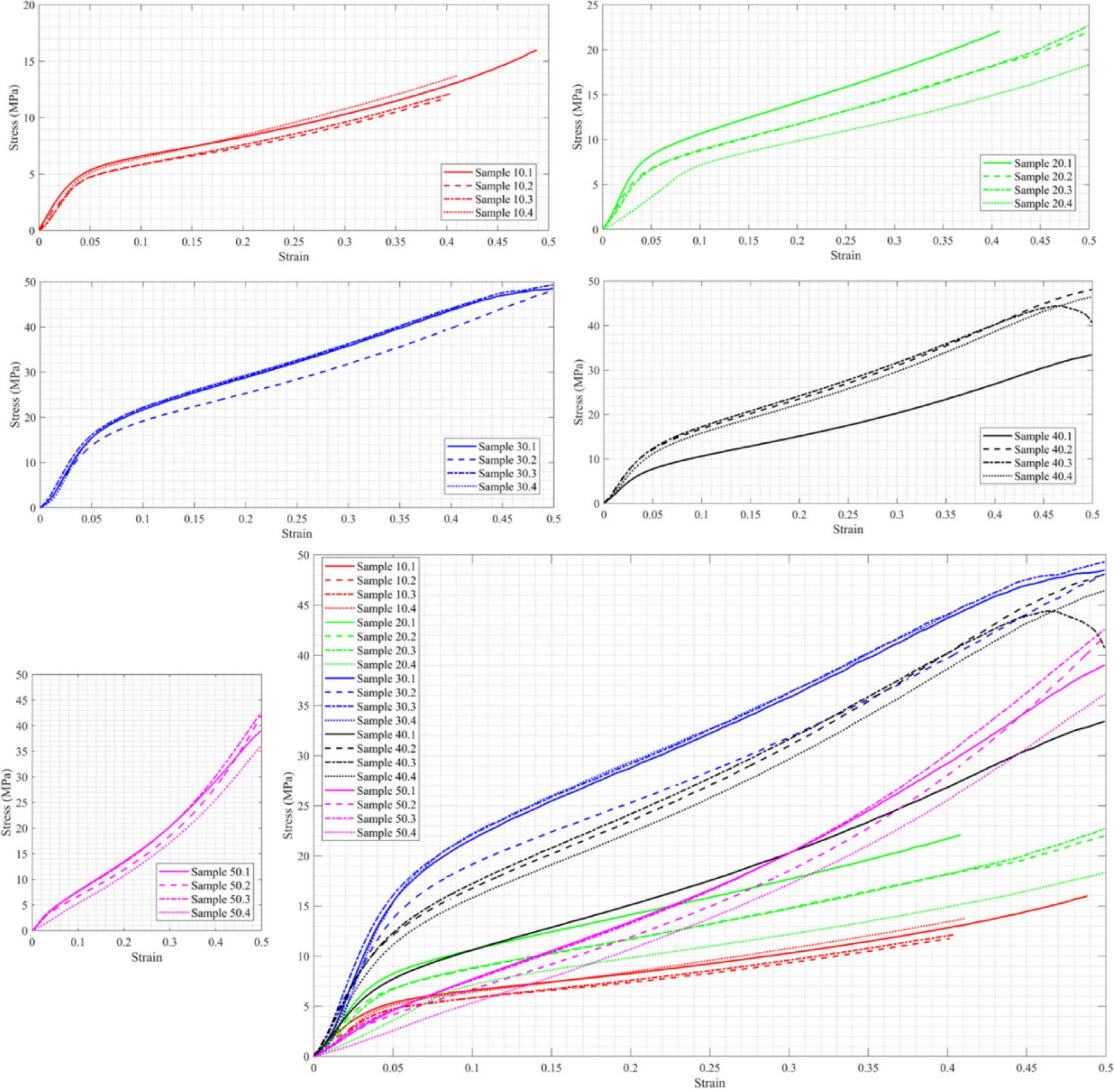
Stress–strain diagram of C10-C50, M2-M1, and all cases together under compressive stress.

The material’s compressive strength varies with the changing ratio of C. Figure 6 show that the maximum compressive strength occurs at C = 30, while the minimum compressive strength is reached when C = 10, and the ratio of M has a minimal effect. In other words, increasing C enhances the compressive strength until C reaches 30%, and the strength improves further as C increases to 50%.

**Fig. 7:**
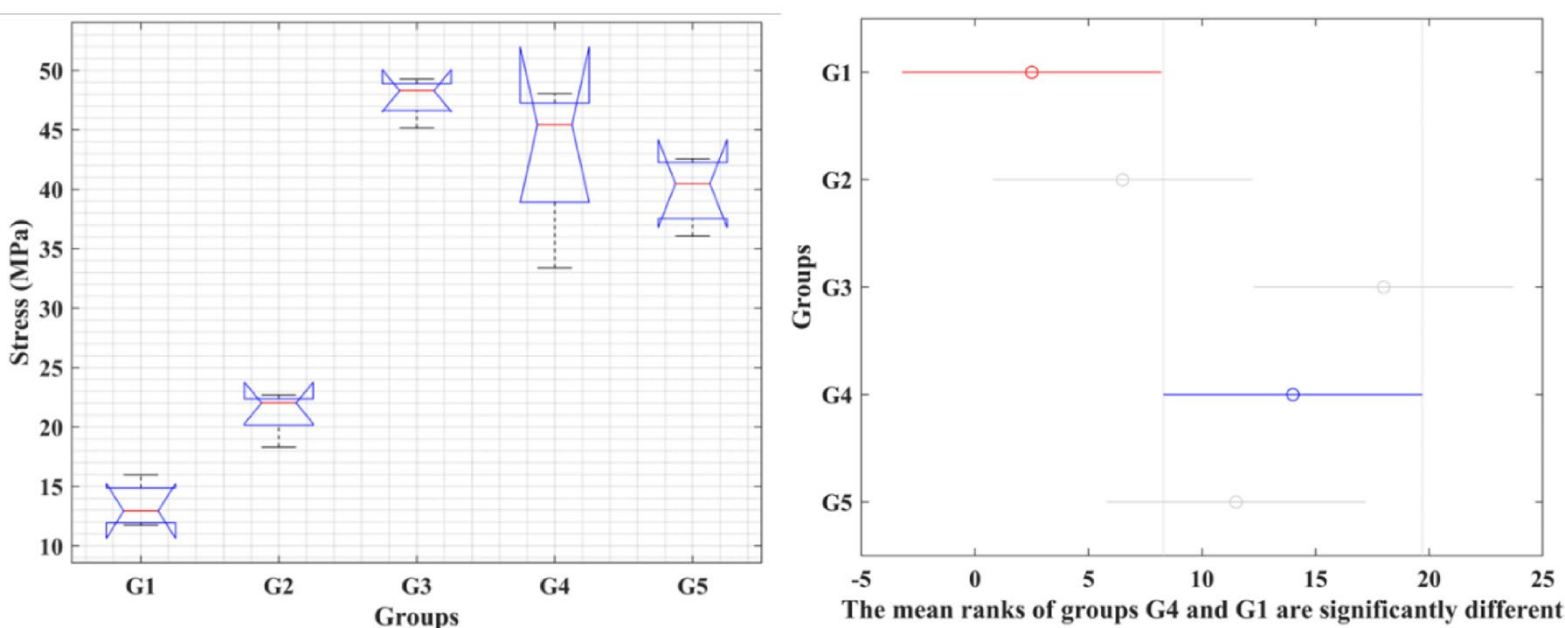
Kruskal–Wallis test for compressive strength of the five groups and the variance of the groups based on the median G4.

This is clear from Fig. 7, which shows that the differences in compressive strength across the five groups are statistically significant, as the p-value is less than 0.05. This means that the impact of cellulose content on compressive strength. G3 and G4 exhibit significantly higher strength compared to G1 and G2. G5 may not show significant differences from G3/G4 or G2, but it falls in between. Figure 7 that if G4 is chosen as the median, then G3 differs from all the others.

#### Comparison with OSB

**Fig. 8:**
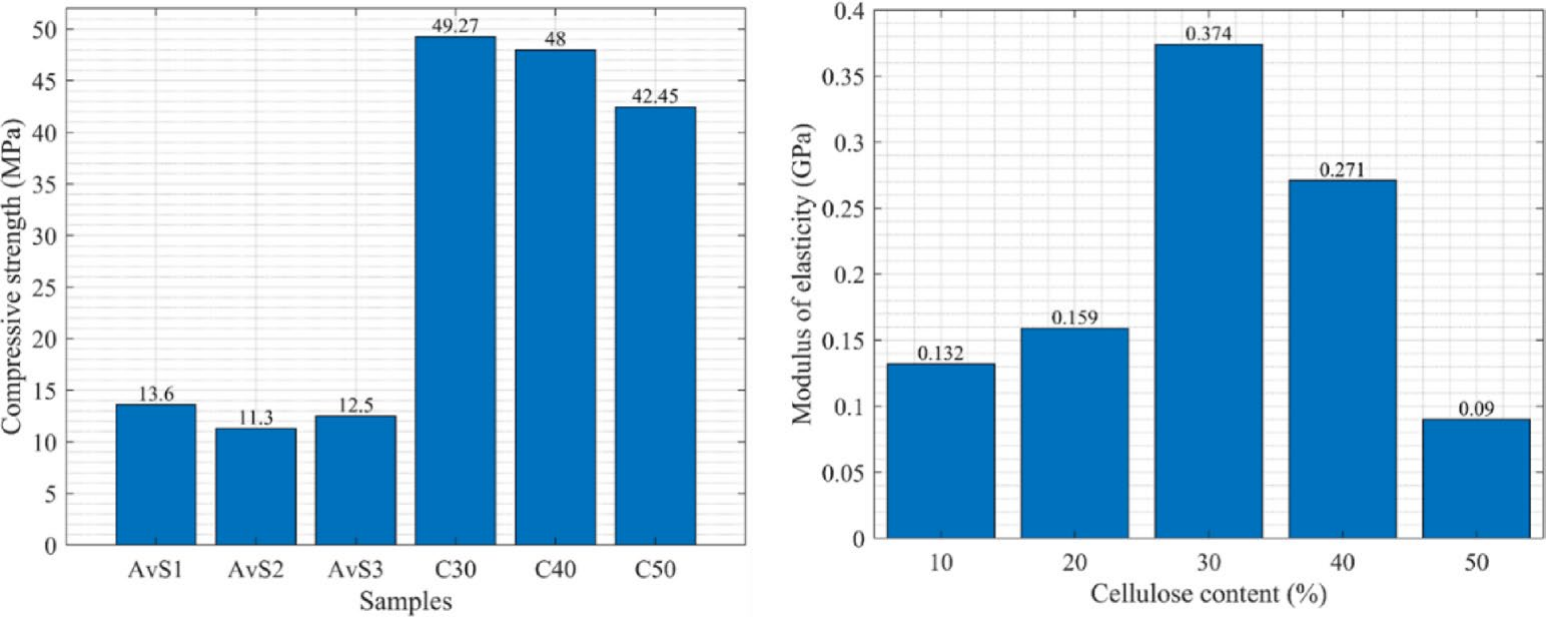
Comparison of the results of compressive strength with OSB^32^, and based on the compressive stress of the samples with varying cellulose contents.

The measured compressive strength values of the cellulose boards are higher than those reported for OSB in the referenced study; nevertheless, differences in material architecture, testing methodology, and intended use preclude direct structural replacement, as shown in Fig. 8. The highest compressive strength among cellulose boards is nearly 50 MPa, while for OSB boards it is 13.6 MPa. Based on the reported values, the tensile and compressive strengths of the prepared cellulose boards compare favorably with or exceed those reported in the literature for OSB. Chen and He^32^.However, due to differences in testing conditions and the small sample sizes, this observation should not be considered statistically significant. Thus, it can be said that comparison serves as a contextual benchmark rather than evidence of direct structural equivalence.

#### Determination of MOE

The results from the compression test on the testing machine were used to calculate the modulus of elasticity (MOE) for each sample. The calculation was carried out similarly in accordance with Hooke’s law. The stress and strain values in the graph’s initial, most linear section were obtained by reading them directly from the graph. Additionally, the average MOE was calculated for each cellulose content, and a graph illustrating this relationship is displayed in Fig. 8.

Data analysis reveals that MOE from compressive tests is significantly less than that from tensile tests. Comparison of Figs. 5 and 8 reveals that even the highest MOE obtained in compression (0.374 GPa) is less than the lowest obtained in tension (0.483 GPa). Furthermore, the data trends are opposite for the two tests. While the tensile test revealed a clear increase in stiffness with increasing cellulose content, the compression test showed no such regular trend. The stiffest samples in compression were those with 30% ground newspaper content, and the least stiff samples were those with 50% ground newspaper content.Also, the role of material architecture in compression has been highlighted in sandwich-structured bio-composites based on agave sisalana plant waste combined with PLA, manufactured using layered manufacturing methods. These materials rely on geometric optimization to improve stiffness and compressive strength rather than matrix reinforcement. By contrast, the current work obtains compressive properties through hot-press-induced densification^34^.

From the above observations, it can be inferred that the newly formed polyurethane-cellulose composite material possesses mechanical anisotropy. The main reason may be the hot-pressing process and the orientation of cellulose particles.The mechanical anisotropy in the polyurethane-cellulose composites can be explained by the combined effects of material processing and the inhomogeneous distribution of the fillers. During hot-pressing, the compressive stresses, together with the polyurethane’s viscous flow, partially align the cellulose particles in the plane of the board. The alignment increases the efficiency of stress transfer during tensile loading in the plane of the board, while compressive loading in the direction perpendicular to the preferred alignment reduces the board’s stiffness and strength. The lack of long fibers in the ground newspaper, together with the random orientation of the latter, increases the localized stresses, which are more accentuated during the compressive loading. The same effects have been observed in the hot-pressed lignocellulosic composites.

To conclude, the analysis of the results showed no clear effect of the amount of cellulose waste on the results, which were several times lower than those under tensile stress. In this respect, it can be concluded that the tested composite is not isotropic. It exhibits better tensile-stress properties than compressive-stress properties. To fully confirm this tendency, it would be worthwhile to test samples of other shapes and cross-sections in future research.

### Impact test

A total of fifteen samples were subjected to the test, three specimens of each type of cellulose waste. Each sample was labeled with the C.M. nomenclature. The whole set of test results is summarized in Table 4. Cross-sectional areas were measured at the fracture point for each specimen. The fracture work K was measured directly from the test machine. The impact strength KT was calculated from this data using the formula:

4 $$KT = \frac{K}{A}$$

**Table 4:** Charpy impact test results.

Pattern	Dimensions of the fracture site			K	KT	KT_śr_	S
	Thickness	Width	A				
[C.M]	[mm]	[mm]	[mm^2^]	[J]	[kJ/m^2^]	[kJ/m^2^]	[kJ/m^2^]
10.1	7.65	11.54	88.28	0.445	5.041	4.857	0.382
10.2	7.64	11.23	85.80	0.379	4.417		
10.3	7.63	11.28	86.07	0.440	5.112		
20.1	7.03	11.07	77.82	0.290	3.726	4.262	0.758
20.2	6.61	9.90	65.44	0.314	4.798		
20.3	8.53	10.64	90.76	0.293	3.228		
30.1	7.42	10.71	79.47	0.404	5.084	4.524	0.493
30.2	7.23	10.69	77.29	0.321	4.153		
30.3	7.45	10.71	79.79	0.346	4.336		
40.1	7.81	10.66	83.25	0.391	4.696	4.859	0.386
40.2	7.72	10.66	82.30	0.377	4.581		
40.3	7.61	10.66	81.12	0.430	5.301		
50.1	7.43	10.78	80.10	0.329	4.108	4.243	0.119
50.2	7.44	10.80	80.35	0.348	4.331		
50.3	8.12	10.82	87.86	0.377	4.291		

**Fig. 9:**
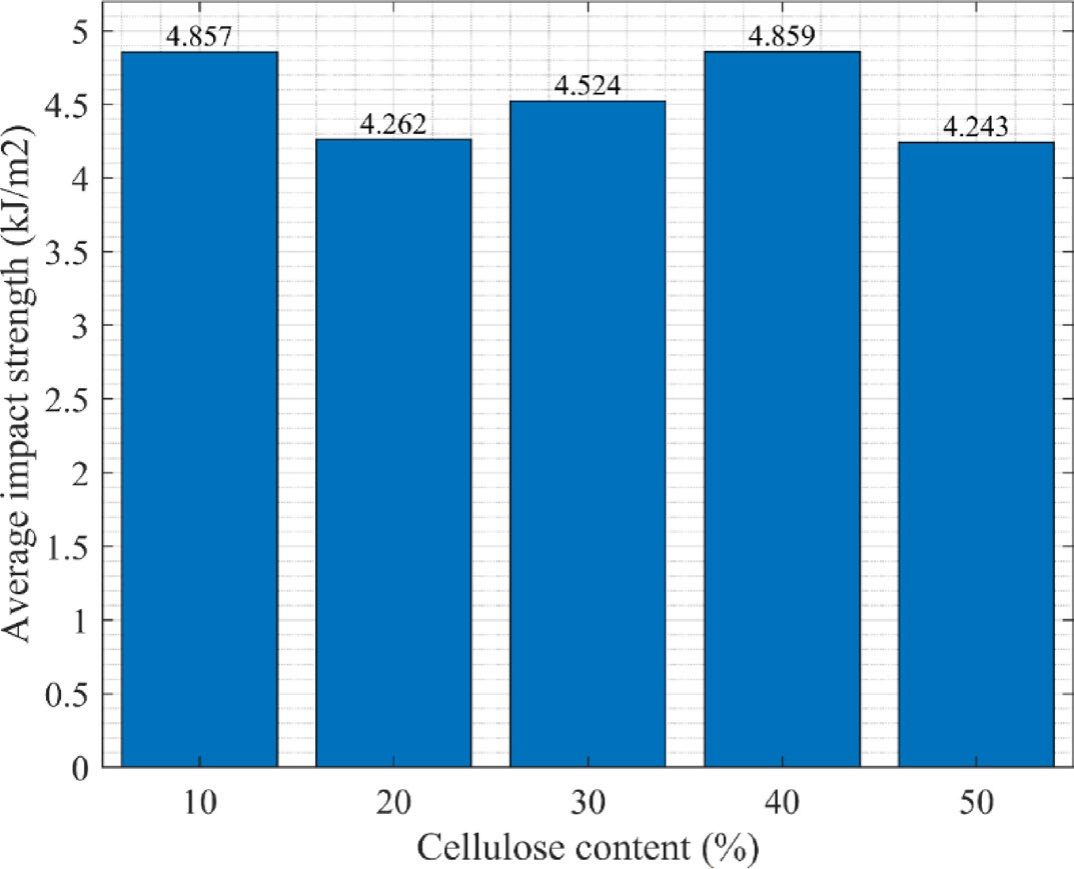
Dependence of impact strength value on cellulose content in the sample.

The average impact strength was determined for each cellulose content level, and the standard deviation (S) was calculated from the individual results. Sample 20.3 was excluded from these calculations and subsequent analyses due to an evident longitudinal defect, which may have significantly reduced its impact strength. Figure 9 illustrates the correlation of cellulose content in the samples with their respective average impact strength.

There is no statistically significant correlation between cellulose and impact resistance. Although some variations are observed across different compositions, they are within the scope of experimental error and are not statistically significant. Charpy impact strength values were between 4 and 5 kJ/m^2^ with no trend, either increasing or decreasing, as the filler content changes. Maximum stability was observed in the 50% ground newspaper samples, while the 20% filler samples showed the highest variability, likely due to the omission of a faulty third specimen and the reliance on only two data points. As shown, the spread between the maximum and minimum impact strength values is of the same order as the calculated standard deviation, indicating little statistical significance. To lend greater credibility to these results, it is suggested that additional specimens be tested to yield a stronger average and a more accurate picture of the material’s impact performance.

The Charpy impact test is a common method for assessing the sudden-impact resistance of materials, primarily metals and plastics. However, details about its application on wood-based materials are uncommon. A helpful comparison is provided in Table 5, which quotes the impact strength of lignofolas 7.0 J/cm^2^ (i.e., 70 kJ/m^2^)^35^, approximately 14 times greater than the figures obtained for the polyurethane–cellulose composite boards. Lignofol, however, is a considerably stronger material, made by high-pressure consolidation of thin wood veneers with synthetic adhesives. Its mechanical performance is thus significantly higher than that of natural wood, making it suitable for structural applications with high demands. Thus, despite lignofol’shigher impact resistance, it cannot serve as a substitute for, or quantify, the composite materials being researched in this work.

**Table 5:** Comparison of the impact resistance of Lignofol and the cellulose board.

Property	Lignofol^35^	Cellulose
Impact strength (kJ/m2)	70	4.859
Compressive strength (MPa)	100	50

In conclusion, the results indicate no clear correlation between the composition of the tested samples and their impact strength. As the proportion of cellulose filler varied, the measured impact strength values consistently remained within the 4–5 kJ/m^2^ range, showing no discernible trend of increase or decrease. This lack of observable dependence is likely influenced by the limited number of samples tested, suggesting that a larger sample size is necessary to improve statistical reliability and accuracy. Compared with materials reported in the literature, the obtained values are several orders of magnitude lower. For instance, lignofol, a high-performance composite engineered for mechanically demanding applications, exhibits significantly higher impact strength. Its superior performance is attributed to a distinct manufacturing process that uses synthetic adhesives, elevated temperatures, and high-pressure pressing. Consequently, lignofol is not directly comparable to the material.

#### Comparison with literature

The mechanical and impact properties of the developed polyurethane-cellulose boards were compared with data reported in similar studies. Hejna et al.^36^ demonstrated that rigid polyurethane-polyisocyanurate foams derived from crude glycerol and castor oil-based polyols exhibited compressive strengths between 0.2 and 0.7 MPa. The materials investigated in this study achieved values exceeding 0.8 MPa, indicating a higher load-bearing potential without the use of commercial catalysts. Similarly, Piszczyk et al.^37^ developed foams based on polyglycerol with comparable density and flexural properties, but without structural integrity required for board production. Moreover, the use of reduced graphene oxide as reinforcement in polyurethane nanocomposites improved thermal and mechanical behavior, suggesting that further optimization could involve functional nanofillers^26^. Compared with prior work, the present results highlight a balance among material simplicity, mechanical performance, and environmental benefits, positioning this system as a viable alternative in bio-based panel technologies.

### Thermogravimetric analysis

**Fig. 10:**
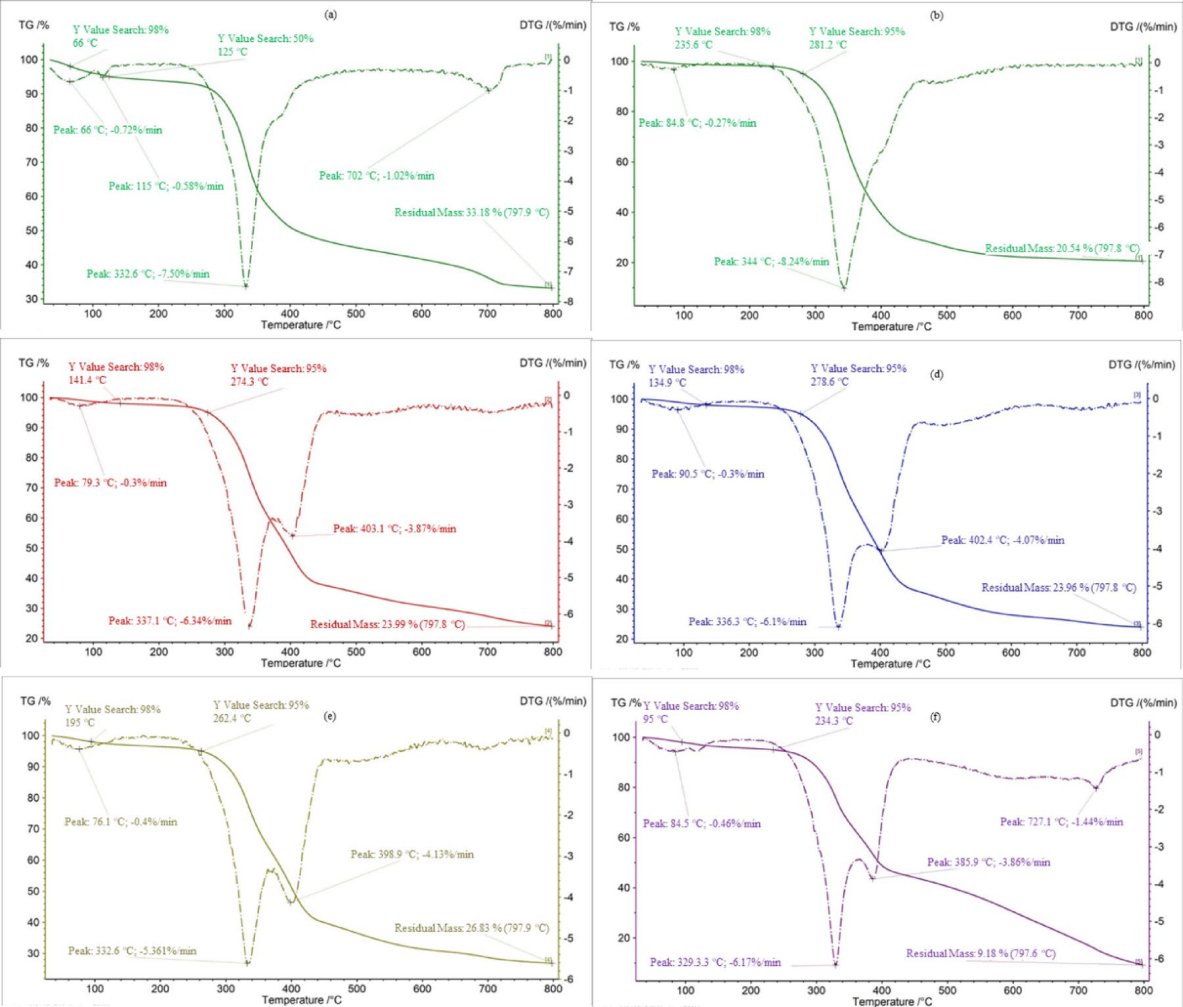
TGA analysis for (**a**) 0%, (**b**) 10%, (**c**) 20%, (**d**) 30%, (**e**) 40%, and (**f**) 50%cellulose waste.

Dynamic TGA was employed, in which mass variation was measured as a function of temperature under a linear heating rate. The results were presented as thermogravimetric (TG) curves and their first derivatives (DTG), plotted against temperature or time, as illustrated schematically in Fig. 10. The TG curve shows the relative mass change of the sample, while the DTG curve shows the rate of mass loss as a function of temperature.

The thermogravimetric analysis in Fig. 10 shows clear trends related to cellulose waste content. Initially, mass loss occurs due to the evaporation of moisture and volatile compounds. Pure cellulose waste exhibits the fastest weight loss, reaching a 98% mass reduction at approximately 66 °C, confirming its high moisture content. Conversely, polyurethane composites with lower cellulose levels retain their mass longer; the 50% cellulose sample reaches 98% loss at approximately 95 °C, while the 10% sample only reaches this point at around 235 °C, indicating a lower moisture content.The main decomposition, linked to pyrolysis and overall weight loss, occurs at different temperatures depending on the composition. Samples with 10% cellulose start to break down rapidly at roughly 250 °C, increasing to about 270 °C for 50% cellulose, and up to 280 °C for pure cellulose. This indicates enhanced thermal stability with higher newspaper content.The residual mass after testing also varies with the proportion of cellulose. The 10% cellulose sample yields about 20.5% residue, while pure cellulose yields about 33.2%. Interestingly, the 50% composite shows only about 9.2% residue, which deviates from the expected trend of approximately 30%. This anomaly suggests that further testing is needed for confirmation.

Table 6 compares the fundamental values characterizing each sample, as read from the TG and DTG graphs. These include: the amount of material remaining after the test, the highest rate of mass loss, and the temperature at which it occurs.

**Table 6:** Characteristic points of TG and DTG graphs from TGA examination.

Cellulose content	Amount of material remaining	Fastest weight loss	Temp. of fastest weight loss
(%)	(%)	(%/min)	(°C)
10%	20.54	− 8.24	344.0
20%	23.99	− 6.34	337.1
30%	23.96	− 6.10	336.3
40%	26.83	− 5.61	332.6
50%	9.18	− 6.17	329.3

**Fig. 11:**
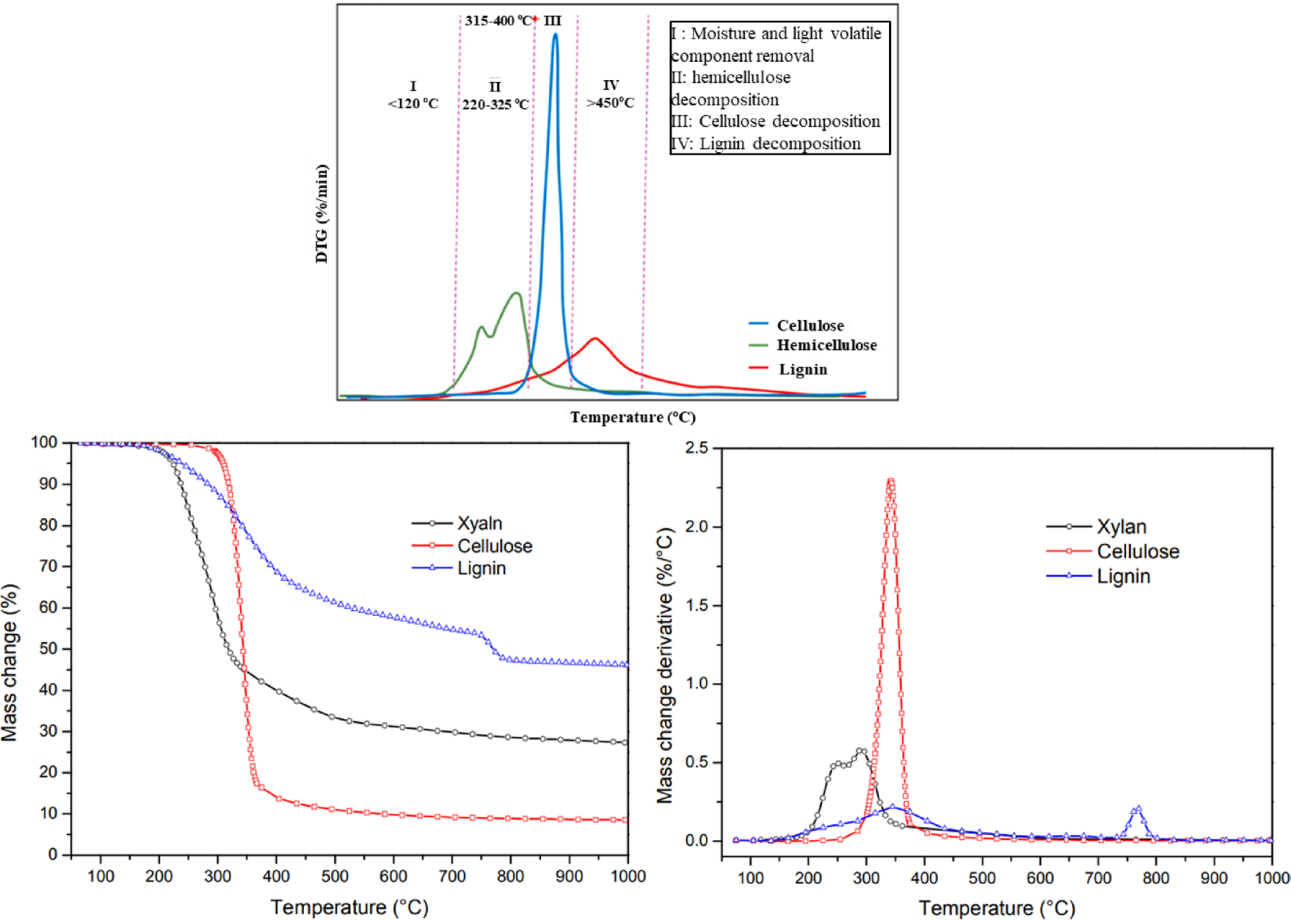
TGA analysis of different types of cellulose^21^, and TG and DTG curves test of different types of materials, including cellulose^39^.

Cellulose, hemicellulose, and lignocellulose were studied^38^ then presented by Escalante et al.^21^. They analyzed the materials under inert conditions to evaluate their combustion potential, with DTG results showing decomposition primarily between 280 and 400 °C, which is similar to the results obtained in the present study for cellulose waste (see Fig. 11). However, unlike the present work, their study lacks TG curves, mass loss rates, and residue data, limiting detailed comparison. In the study^39^, cellulose, hemicellulose, and lignin were analyzed using TGA (NETZSCH STA 449F) under nitrogen with a 10 °C/min heating rate up to 1000 °C, and TG/DTG curves were reported as presented in Fig. 11. Although additional fast and slow pyrolysis tests were conducted with a custom macro-TGA system, only the conventional TGA results are relevant for comparison. The use of recycled polymeric and filler materials has also been considered in the fused deposition modeling of recycled polypropylene and aluminum powder, in which improvements in stiffness and thermal properties were achieved, though constrained by the filler material’s dispersibility. The current study also shows that the use of recycled material can yield a stable composite when processing conditions are well optimized^40^.

### Water vapor permeability test

**Fig. 12:**
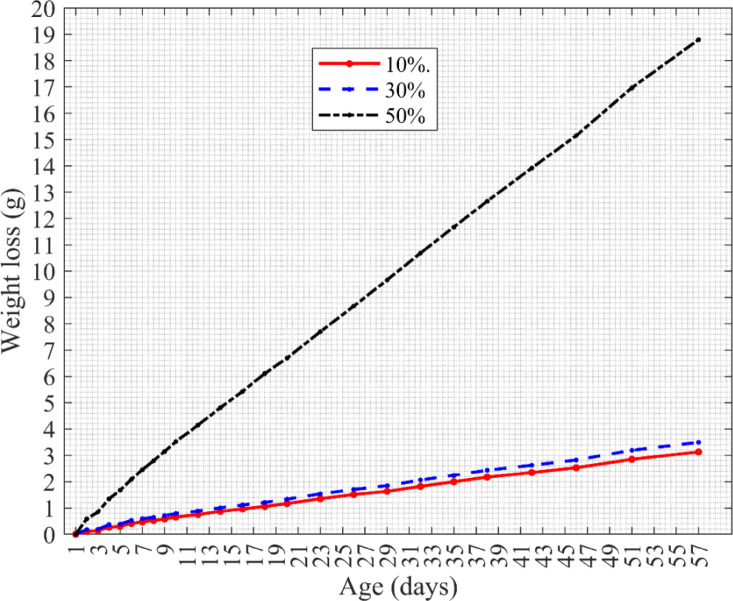
Average weight loss of test sets with samples containing 10%, 30%, and 50% of cellulose.

The study used the wet-vessel method, in which a saturated aqueous solution maintained a constant water–vapor pressure inside the test vessel. Due to the partial pressure difference between the vessel and the desiccant chamber, water vapor moved through the permeable samples and was absorbed by the desiccant. Periodic weighing of the test samples allowed determination of vapor mass transfer under steady-state conditions, as indicated by a linear loss of mass over time. Initially, daily weighing was conducted, then intervals were gradually extended to reduce disturbances from opening the chamber, which was sealed with lithium grease to ensure airtightness. Measurements were taken over 57 days, and the results were analyzed. The average test results for the five samples at each cellulose content were calculated and presented in Fig. 12. The graphical analyses showed total mass change, relative mass loss, and daily vapor transfer as functions of cellulose content. The data showed large initial fluctuations, then stabilized after approximately 10 days.

The test results showed that water vapor generated by the saturated solution penetrated the permeable specimens and was absorbed by the desiccant, resulting in a gradual decrease in mass of the sets. After approximately 10 days, the mass change of the samples became linear, indicating a steady flow of water vapor. Increasing the cellulose content, introduced as ground newspapers, led to higher vapor transmission. Comparative measurements further demonstrated that the polyurethane–cellulose composites allowed greater vapor flow than conventional wood-based panels. Specifically, OSB and MDF boards exhibited vapor permeability values approximately 7 times lower than those of the tested composites.

To further contextualize the obtained results, Table 7presents a comparative overview of the mechanical and selected physical properties of the developed polyurethane–cellulose composites in relation to conventional wood-based panels and representative bio-based composite materials reported in the literature.

**Table 7:** Comparative overview of the mechanical properties of the developed polyurethane–cellulose composites and selected conventional and bio-based composite boards reported in the literature. Values are presented as representative ranges due to differences in material density, specimen geometry, and applied testing standards.

Material	Density (kg/m^3^)	Tensile strength (MPa)	Compressive strength (MPa)	MOE (GPa)	Impact strength (kJ/m^2^)	References
Polyurethane–cellulose composite (this study, C30–C50)	n/a	up to ~ 13	up to ~ 50	0.5–1.2	higher than MDF (trend)	This study
Oriented Strand Board (OSB)	600–700	5–10	10–15	3–5	moderate	Chen and He^27^
Medium Density Fiberboard (MDF)	700–800	10–20	15–25	2–4	low	EN 622–5
Bio-based polymer composite (natural fiber reinforced)	900–1200	20–40	20–60	2–6	moderate to high	Faruk et al. (2012)
Waste-derived composite board (polymer-based)	500–900	8–25	15–40	1–3	moderate	Koronis et al. (2013)

### Dynamic analysis (DMA)

This section describes a study in which the boards underwent dynamic analysis (DMA). The theoretical analysis procedure and the results for each cellulose filler content in the sample will be discussed. DMA is a method for determining a material’s viscoelastic properties. It also allows determination of the temperature dependence of the dynamic modulus of elasticity, E*, and its two components: the storage modulus and the loss modulus. During DMA testing, a sinusoidal deformation is imposed on the test sample, and the resulting stress is measured. The dynamic modulus of elasticity E* is determined based on the stress–strain relationship obtained during the test.

All evaluated parameters exhibited comparable trends across cellulose contents. Increasing the cellulose fraction enhanced the mechanical response, particularly through a substantial rise in the storage modulus, and consequently, in the dynamic modulus of elasticity. The DMA results demonstrated that the storage modulus of the sample with the highest cellulose content (50%) exceeded that of the sample with the lowest cellulose content (10%). Similarly, the loss modulus increased with the cellulose fraction, indicating that the highly filled material dissipates more energy than the low-filled counterpart. Compared with the 10% sample, the 50% cellulose-filled board exhibited markedly improved energy and vibration absorption.

The temperature-dependent behavior of the cellulose boards is summarized as follows:

- Storage modulus: A measure of material stiffness, it decreased with increasing temperature, which is characteristic of polymeric systems, as elevated temperature typically reduces rigidity.
- Loss modulus: Representing the energy dissipated through internal friction, it reached a maximum in the range of 50–70 °C and subsequently decreased with further heating, reflecting a reduction in material viscosity.
- Tan delta (damping factor): The damping factor reached a maximum between 100 and 115 °C (with the exception of the 50% cellulose-filled samples), after which it decreased for all compositions. This suggests diminished vibration-damping capability at elevated temperatures. The obtained tan delta values (0.01–0.4) indicate a tendency toward brittle behaviour, which was corroborated by tensile strength tests.

**Fig. 13:**
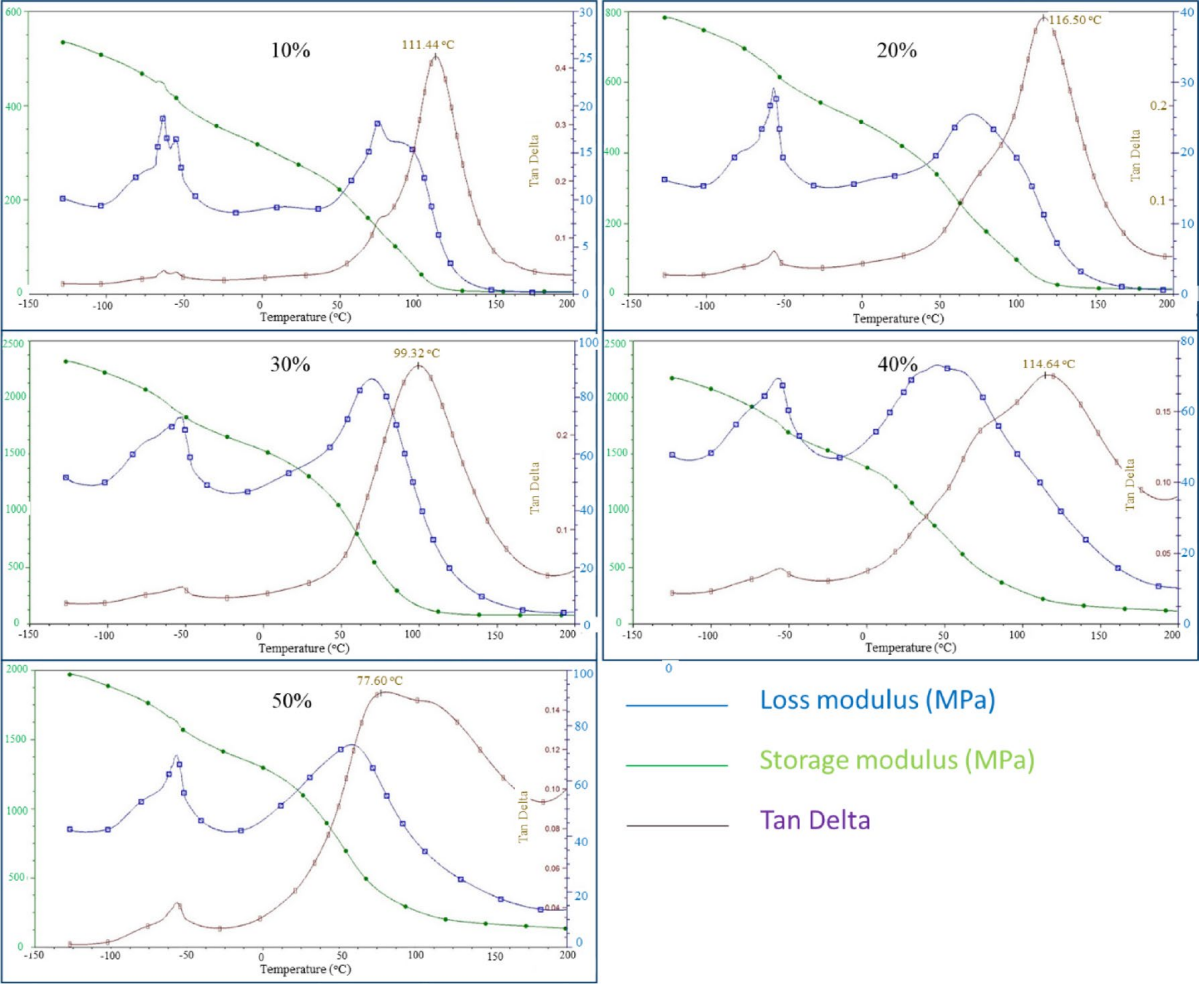
DMA analysis graph for a composite with various contents of cellulose waste.

Based on the DMA studies presented in Fig. 13, it can be concluded that a higher cellulose content increases the material’s strength parameters the storage modulus and dynamic modulus of elasticity significantly increase, thereby also enhancing the material’s ability to absorb energy and vibration. These are desirable values and increase the potential for using the new material in construction, such as building partitions. The ecological aspect is also met, as a larger amount of waste can potentially be reused. Similar increases in storage modulus have been reported in 3D-printed PLA composites with strategic cores composed of sisal fibers, in which the relationships between stiffness and alignment, as well as core-skin interactions, are strongly emphasized. Contrary to architected composites, the stiffness development in the current boards results from increased filler content and densification^41^.

## Conclusions

This study proposes using a construction element made from recycled waste paper as an environmentally friendly construction material widely used in industry. The study revealed the physical and mechanical properties of the prototype material under different loadings. It was found that.

- Increasing cellulose content boosts tensile strength.Adding cellulose filler at a 30% sustaining level resulted in reasonable tensile strength.
- The 30% cellulose achieves the highest compressive strength.
- The Modulus of Elasticity (MOE) determined from the tensile and compressive tests for the 30% cellulose content was 1.235 and 0.374, respectively.
- A comparison between the proposed material and OSB revealed that tensile strength values for C30 and C50 are similar to or greater than those reported in the literature for OSB under tensile stress. However, this does not imply direct substitution in structural applications
- The proposed cellulose board exhibits greater ductility than OSB under tensile and compressive stresses.
- The cellulose boards exhibit much lower compressive strength compared to tensile strength.
- Regarding the materials’ resistance to impact load, cellulose content does not significantly affect it; the samples exhibit similar resistance to impact loads.The proposed material has 14 times less impact resistance than lignofol.
- The fastest weight loss occurs at the temperature of 30–45 °C, which is also comparable to the studies in the literature
- The tested polyurethane–cellulose composites exhibited higher vapor transmission with increasing cellulose content but showed significantly lower resistance to water vapor compared to OSB and MDF boards.
- DMA studies show that higher cellulose content enhances strength, energy absorption, and vibration damping, while also enabling greater reuse of waste. These properties qualify the material as a promising candidate for specific non-load-bearing and lightly loaded construction applications, rather than as a direct substitute for conventional structural wood-based panels.
- The polyurethane-cellulose composite that we propose is likely to be a viable replacement for OSB only in applications that are designed to benefit from the low-density nature, sustainable qualities and excellent physical performance attributes of such composites; therefore, examples of target applications would include lightweight modular panels, interior partitions, and insulating construction components.

The results of this study indicate the feasibility of producing rigid polyurethane boards from recycled newspaper-derived polyols without the addition of commercial catalysts. The composite materials exhibited favorable mechanical and impact resistance, as well as low density and dimensional stability. These properties qualify them as promising candidates for use in interior building applications such as lightweight partition panels, modular wall systems, and insulating construction elements. The use of recycled raw materials and simplified synthesis aligns with current trends in low-emission construction and circular economy practices. Further research may explore scale-up potential, long-term durability, and fire resistance performance to meet specific industry standards.

### Future work recommendation

Future studies should focus on characterizing the microstructure, including scanning electron microscopy (SEM), to evaluate the dispersion of cellulose particles, interface bonding, and preferential orientation induced by the hot-pressing process.

## Supplementary Information

Below is the link to the electronic supplementary material.

Supplementary Material 1

## Data Availability

The data that support the findings of this study are available from the corresponding author upon reasonable request.
