# Supplementary material for: Recycling paper waste into structural cellulose composites with enhanced mechanical and thermal performance
Source: Sci Rep. 2026 Mar 21;16:14384. doi: 10.1038/s41598-026-43032-7 (PMC13144366; doi:10.1038/s41598-026-43032-7)
Supplement: Supplementary file 1 — Supplementary Material 1 [file 41598_2026_43032_MOESM1_ESM.docx]

# Appendix


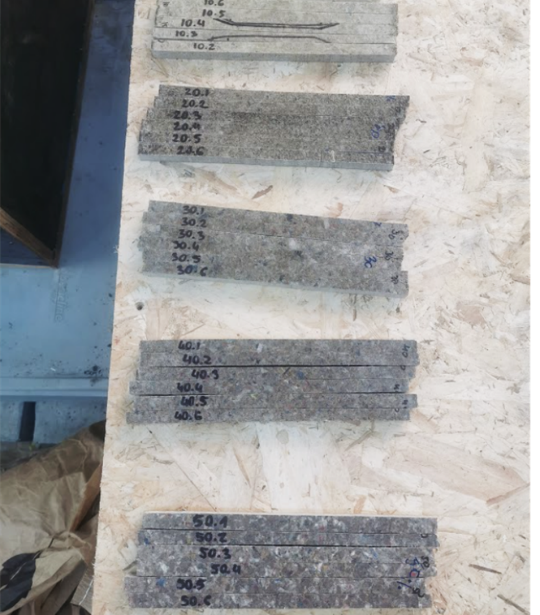


Figure 14 Casted samples for the tensile strength test


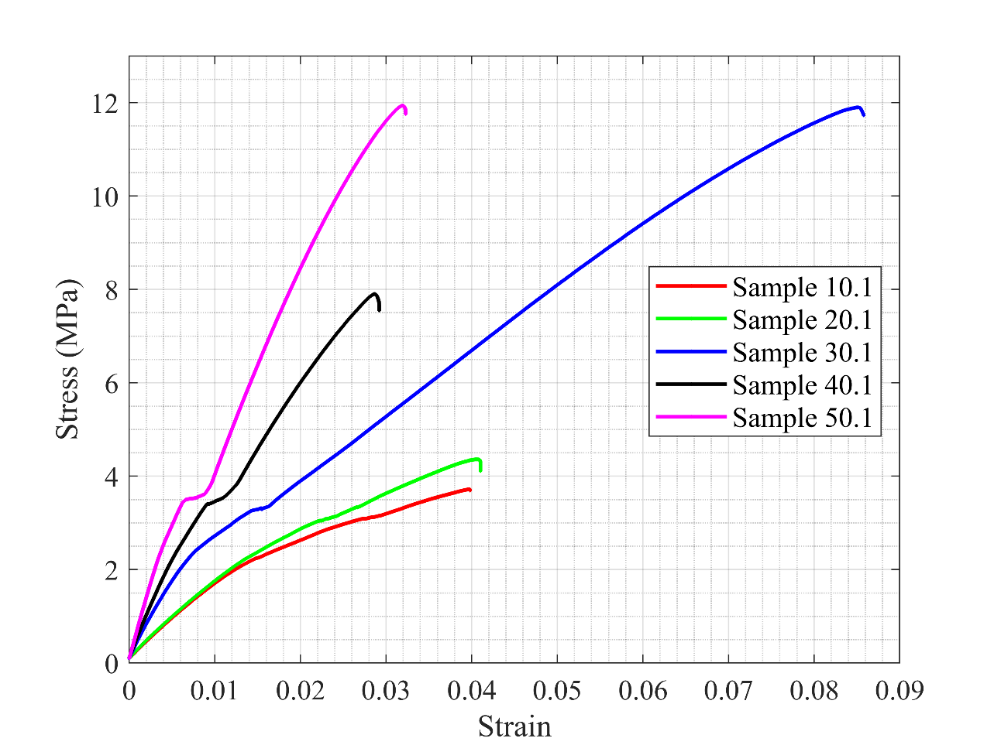


Figure 15 Control stretching of samples - force-elongation graph


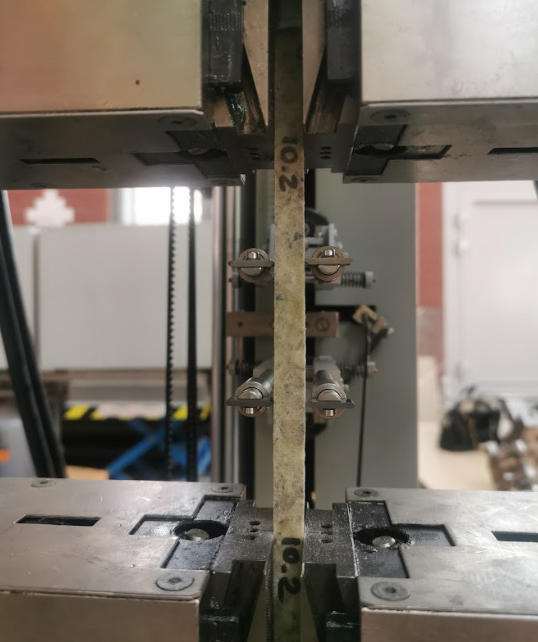


Figure 16 Sample No. 10.2 after applying the initial force and before starting the main test


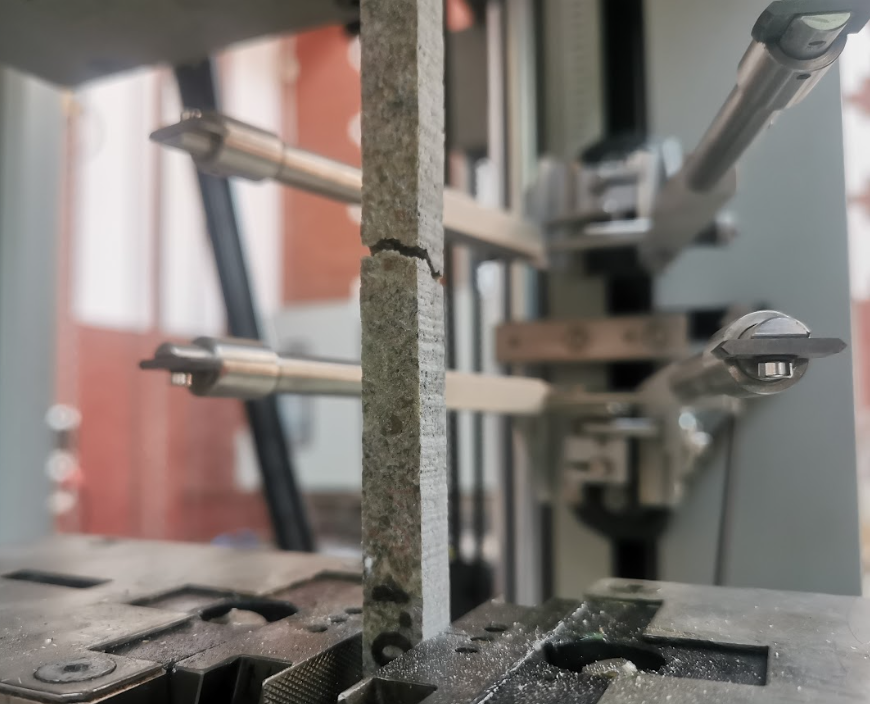


Figure 17 Brittle failure of the sample – example no. 1


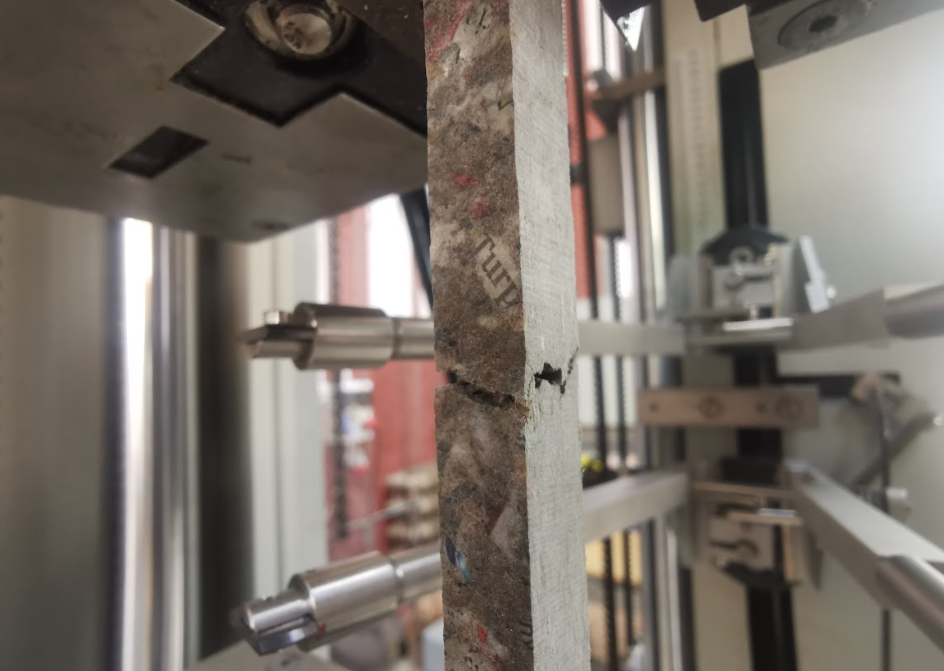


Figure 18 Brittle failure of the sample – example no. 2


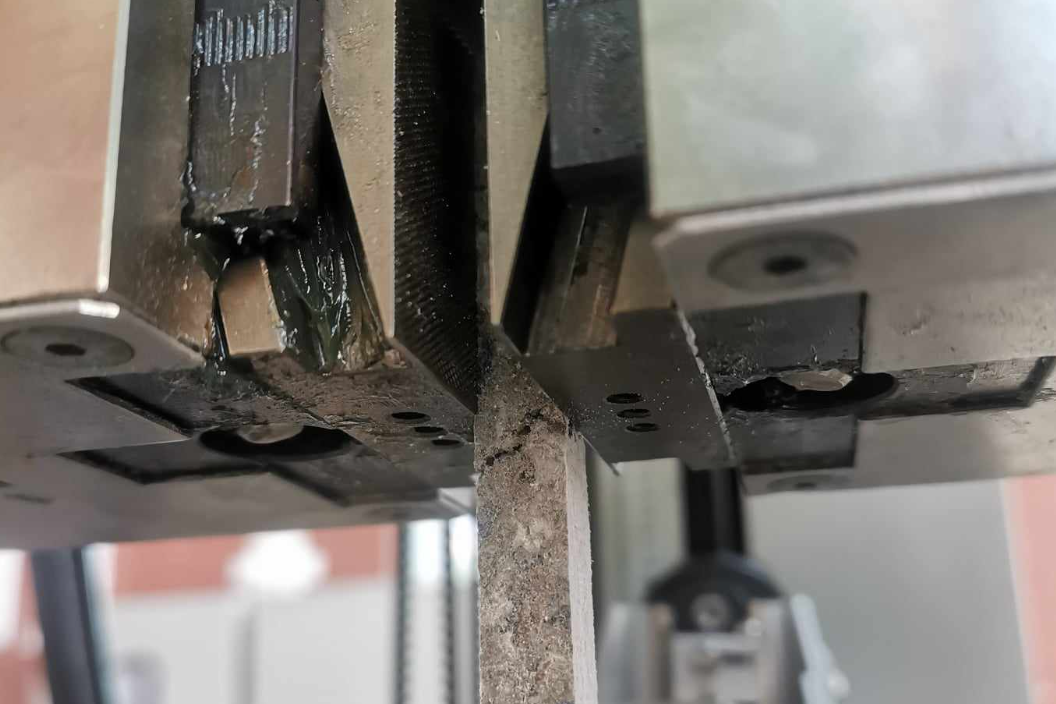


Figure 19 Destruction of sample no. 30.3 in the clamping zone in the jaws


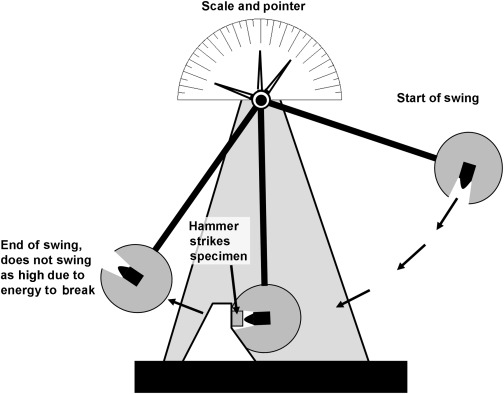


Figure 20 The Charpy test schematic diagram source: [42]


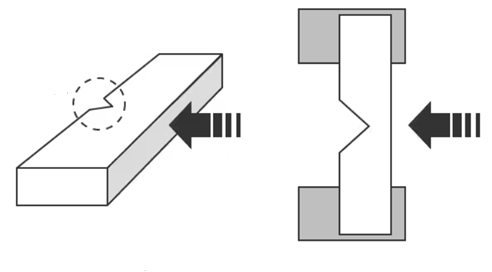


Figure 21 Test sample with V-shaped cutout source:[42]


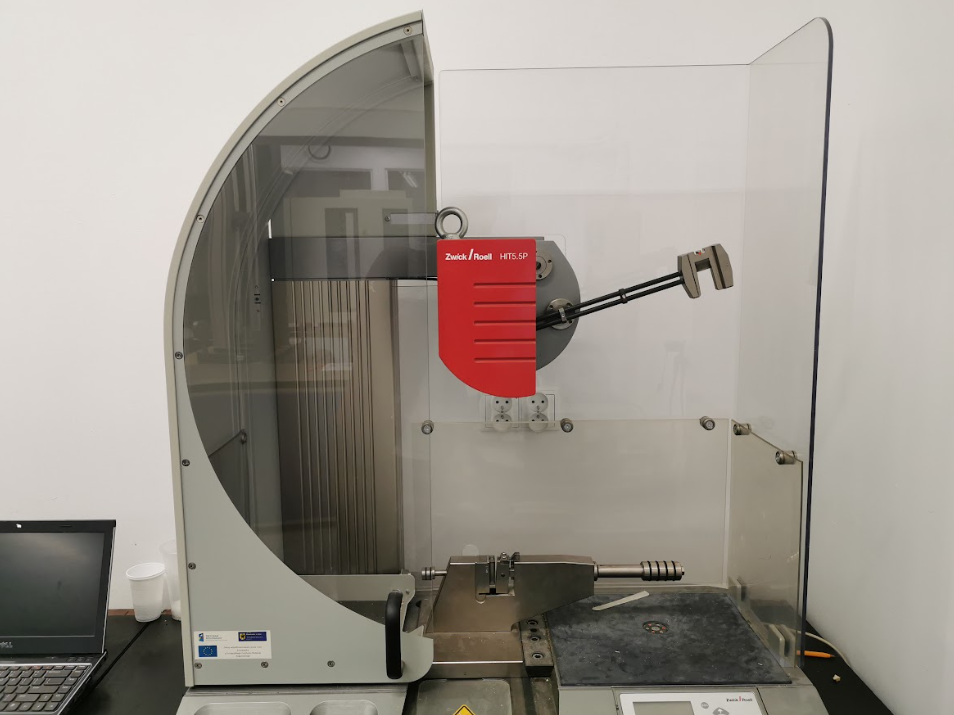


Figure 22 Zwick Roell HIT 5.5P testing machine with a 2.0 J hammer mounted


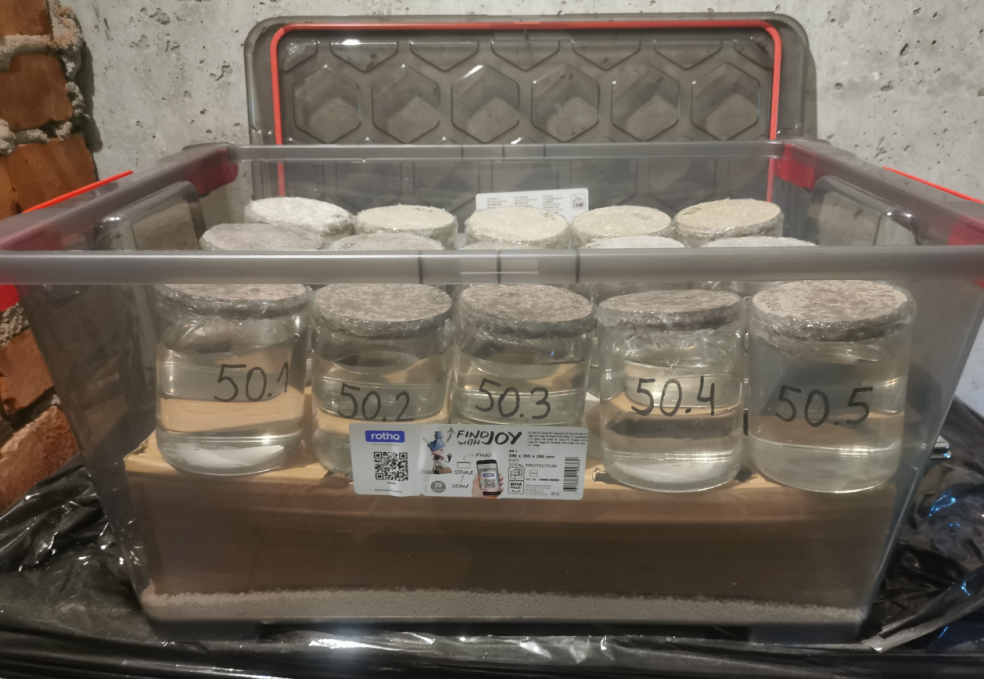


Figure 23 Test kits placed in the test chamber - side view

Table 8 Summary of cross-sectional dimensions of samples in the tensile test

| Sample (C.M) | X(mm) | Y(mm) | Area(mm^2^) |
| --- | --- | --- | --- |
| 10.1 | 10.8 | 10.3 | 111.24 |
| 10.2 | 10.7 | 9.8 | 104.86 |
| 10.3 | 11.0 | 7.7 | 84.70 |
| 10.4 | 10.8 | 10.0 | 108.00 |
| 10.5 | 10.7 | 9.4 | 100.58 |
| 20.1 | 10.4 | 10.0 | 104.00 |
| 20.2 | 10.4 | 9.6 | 99.84 |
| 20.3 | 10.8 | 9.9 | 106.92 |
| 20.4 | 10.5 | 9.9 | 103.95 |
| 20.5 | 10.5 | 10.0 | 105.00 |
| 30.1 | 10.8 | 9.6 | 103.68 |
| 30.2 | 10.8 | 9.0 | 97.20 |
| 30.3 | 10.8 | 9.9 | 106.92 |
| 30.4 | 10.8 | 8.8 | 95.04 |
| 30.5 | 10.8 | 9.6 | 103.68 |
| 40.1 | 10.7 | 9.8 | 104.86 |
| 40.2 | 10.6 | 9.3 | 98.58 |
| 40.3 | 10.7 | 9.0 | 96.30 |
| 40.4 | 10.6 | 9.8 | 103.88 |
| 40.5 | 10.6 | 10.2 | 108.12 |
| 50.1 | 10.8 | 10.6 | 114.48 |
| 50.2 | 10.8 | 8.2 | 88.56 |
| 50.3 | 10.8 | 10.2 | 110.16 |
| 50.4 | 10.9 | 8.8 | 95.92 |
| 50.5 | 10.9 | 9.8 | 106.82 |

Table 9 Cross-sectional areas of the samples in the compression test

| Samples C.M | Cross-sectional area (mm2) |
| --- | --- |
| 10.1 | 115.4 |
| 10.2 | 117.8 |
| 10.3 | 116.7 |
| 10.4 | 120.9 |
| 20.1 | 122.0 |
| 20.2 | 123.3 |
| 20.3 | 122.5 |
| 20.4 | 121.0 |
| 30.1 | 121.0 |
| 30.2 | 120.6 |
| 30.3 | 121.2 |
| 30.4 | 121.4 |
| 40.1 | 125.6 |
| 40.2 | 127.3 |
| 40.3 | 127.8 |
| 40.4 | 125.2 |
| 50.1 | 124.5 |
| 50.2 | 124.1 |
| 50.3 | 128.1 |
| 50.4 | 125.1 |

Table 10 The amount of material taken for TGA testing

| Cellulose content | Quantity  [mg] |
| --- | --- |
| 10 % | 9.05 |
| 20 % | 9.06 |
| 30 % | 10.82 |
| 40 % | 10.41 |
| 50 % | 10.88 |
